# Treatment of Multiple Myeloma in Elderly Patients: A Review of Literature and Practice Guidelines

**DOI:** 10.7759/cureus.3669

**Published:** 2018-12-01

**Authors:** Suresh Manapuram, Hamza Hashmi

**Affiliations:** 1 Internal Medicine, Saint Francis Hospital and Medical Center, Grand Island, USA; 2 Oncology, University of Louisville School of Medicine, Louisville, USA

**Keywords:** multiple myeloma, elderly, stem cell transplant

## Abstract

Multiple myeloma (MM) is a clonal disorder of malignant plasma cells that comprises approximately 10% of hematologic malignancies. With median age of 66 at the time of presentation, multiple myeloma is predominantly a disease of the elderly. The availability of new combination regimens and the enhanced safety of autologous hematopoietic stem cell transplant has increased the treatment options for elderly patients with multiple myeloma. We provide a summary of data supporting the current management of elderly patients with newly diagnosed multiple myeloma.

## Introduction and background

Multiple myeloma (MM) is a clonal disorder of malignant plasma cells that comprises approximately 10% of hematologic malignancies. The median age at diagnosis is 66 years, making it predominantly a disease of the elderly. Multiple myeloma accounts for approximately 1%-2% of all cancers and slightly more than 17% of hematologic malignancies in the United States [1]. The annual incidence of MM in the United States is approximately four to five per 100,000. The incidence increases with age and combined with the worldwide increase in the elderly population, there is an anticipated 77% increase in the number of patients older than 65 years diagnosed with MM each year by 2030 [2-4]. Development of newer therapeutic agents and improving supportive care over the last two decades has significantly improved the outcome of MM in younger patients [5-7]. However, most studies suggest that improvements are marginal in elderly patients (defined as age 75 years and older) [2, 6]. This may be explained by the higher incidence of more severe disease in the older patients, but it is mainly due to patient characteristics (e.g. performance status, comorbidities) and organ dysfunction associated with aging [5-7].

Patients older than age 75 are at increased risk of frailty, vulnerable to adverse events associated with high-dose chemotherapy and they are rarely candidates for high dose therapy (HDT) plus autologous stem cell transplant (ASCT). However, the availability of new front-line treatment regimens based on the novel agents thalidomide, bortezomib, and lenalidomide have dramatically changed the management of MM in transplant-ineligible patients. In addition, the goals of therapy in the elderly patients may differ from younger patients; older adults are more likely to prioritize symptom control, prolonged treatment-free intervals, and good quality of life over prolonged survival [2, 8, 9]. In this review, we provide a summary of data supporting the current management of elderly patients with newly diagnosed multiple myeloma.

## Review

Therapy for non-transplant eligible patients

The alkylating agent plus corticosteroid combination with melphalan and prednisone (MP) was the standard of care for over 30 years. The addition of novel agents to MP has provided several therapeutic options for ASCT-ineligible patients with MM.

Thalidomide-based regimens

The first novel agent to be combined with MP was thalidomide. Six randomized trials compared MP with MP plus thalidomide (MPT) as primary treatment in elderly patients [10-15]. The response rates and depth of response were significantly increased in the MPT arm in all six studies (overall response rate of 59% for MPT vs. 37% for MP; P<0.001) [16, 17]. The partial response (PR) rate was 42%-76% vs. 28%-48% with MPT and MP, respectively, and progression-free survival (PFS) was 14-28 versus 10-19 months. Overall survival (OS) was significantly improved in the MPT arm in three out of six studies. A meta-analysis of the pooled data from the six MPT trials showed that addition of thalidomide to MP as a front-line regimen in elderly MM patients is associated with a significant improvement in PFS (5.4 months of benefit; hazard ratio (HR) of 0.67; confidence interval (CI) of 0.55-0.80) and a near significant improvement in OS (6.6 months of benefit; HR of 0.82; CI of 0.66-1.02) [16]. Based on these studies, the combination of MP with thalidomide improves response rates and PFS, but with increased toxicity.

The combination of thalidomide and dexamethasone (TD) was compared with MP in a randomized study in elderly MM patients. While the response rate, including complete response (CR) rate, was superior in the TD arm, there was no benefit in terms of PFS and OS and was significantly shorter in the TD arm, particularly in patients over 75 years of age [18, 19]. These results were explained by higher toxicities and early mortality.

Another randomized study compared the efficacy and safety of cyclophosphamide, thalidomide and dexamethasone (CTD) with MP in elderly patients [20]. CTD improved response rates compared with MP; however, PFS and OS rates were similar with the two regimens. CTD was also associated with higher rates of thromboembolic complications and infections.

Lenalidomide-based regimens

Lenalidomide, thalidomide’s next generation analog, is more potent and less toxic than thalidomide. A randomized phase III trial compared MP versus a combination of melphalan, prednisone, and lenalidomide with or without lenalidomide maintenance (MPR or MPR-R) [21]. Preliminary results of the study showed MPR-R was significantly superior to MP with higher response rates (77% vs. 50%) and greater CR rates (18% vs. 5%), superior to MPR in terms of PFS [2, 21-23]. The toxicity profile is less when compared with MPT and the common adverse events observed were neutropenia, thrombocytopenia, and infections.

A randomized Eastern Cooperative Oncology Group (ECOG) study compared lenalidomide and high-dose dexamethasone (40 mg for four days on, four days off, Revlimid dexamethasone [high dose] (RD)) with lenalidomide and low-dose dexamethasone (40 mg once per week, Revilmid dexamethasone [low dose] (Rd)) in newly diagnosed MM patients [24]. The Rd group was superior to RD with a median PFS of 25.3 months and 87% two-year OS. The RD regimen was associated with higher overall response rate (ORR) than Rd; however, this improvement in response came at the expense of increased toxicity. This trial has been the basis for current use of low dose dexamethasone in almost all patients with MM.

In the Frontline Investigation of Lenalidomide plus Dexamethasone versus Standard Thalidomide (FIRST) trial involving 1623 newly diagnosed MM patients who were transplant ineligible, patients were randomly assigned to MPT for 12 cycles (approximately 17 months), Rd for the same fixed period, and Rd continuously [25]. This trial showed a significant increase in PFS with continuous Rd compared with MPT or Rd for 18 cycles (25.5 months for continuous Rd, 20.7 months for 18 cycles of Rd, and 21.2 months for MPT). Four-year OS was significantly superior for continuous Rd over MPT [5, 25].

Bortezomib-based regimens

Bortezomib, the first-in-class proteasome inhibitor, is another important option for frontline therapy in elderly patients with MM. A large phase III randomized trial called Velcade as Initial Standard Therapy in Multiple Myeloma (VISTA) compared MP and bortezomib plus MP (MPV) in a series of 682 newly diagnosed MM patients [26]. MPV was significantly superior to MP in ORR (71% vs. 35%), CR rates (30% vs. 4%). An updated analysis of the data with a median follow-up of five years demonstrated significant OS benefit with MPV vs. MP, with 13.3 month increase in the median OS (56.4 months vs. 43.1 months) [27]. Based on these data, MPV is recognized as standard of care regimen for use in elderly patients. However, safety was a concern in elderly patients; in the MPV arm, the incidence of grade 3 or grade 4 peripheral neuropathy was 14%, and 30% of patients had to discontinue treatment or at least discontinue bortezomib because of treatment-related adverse events [26].

A reduction in bortezomib toxicity with equal efficacy was demonstrated for weekly (rather than twice weekly) once dosing in the following trials [28-30]. In a Spanish study with bortezomib, melphalan, and prednisone (VMP) induction with reduced intensity bortezomib followed by maintenance therapy with bortezomib-thalidomide (VT) or bortezomib-prednisone (VP), the results showed that after six cycles, the incidence of grade 3 or 4 neuropathy dropped to 7% with an ORR of 80% (20% of CR) [5, 30]. In another study, VMP induction (nine cycles) without maintenance was compared with VMP plus thalidomide (VMPT) followed by maintenance therapy with VT [22, 31]. In both the arms, weekly bortezomib was associated with significantly decreased frequency of severe neuropathy. VMPT-VT improved response rates and PFS [31].

Carfilzomib-based combinations

Carfilzomib, a second-generation proteasome inhibitor, emerged as a new therapeutic option for newly diagnosed transplant-ineligible MM patients. In a pilot phase I/II study, carfilzomib was combined with MP (KMP) and the results are promising with good response rates and an acceptable toxicity profile. Randomized trials comparing KMP with VMP are currently running and the results are awaited [32]. In another study, a combination of carfilzomib with cyclophosphamide and low dose dexamethasone (KCydex) has been evaluated in a series of 53 newly diagnosed elderly MM patients, resulting in an ORR rate of 91% and a CR rate of 47%. The regimen was safe and well-tolerated with no grade 3 or 4 neuropathy reported [33].

Current role of HDT plus ASCT in elderly patients

The great majority of randomized controlled trials comparing HDT plus ASCT and conventional-dose chemotherapy have excluded adults older than age 65 years [34]. However, ASCT is feasible in patients older than 65 years, at least in selected patients with good performance status and no severe comorbidities. Over the past two decades, the proportion of adults older than 70 years undergoing ASCT increased more than five-fold, from less than 1% to 5% [2, 35]. Lower doses of melphalan (100-140 mg/m2) are used for patients older than 70 years. Mehta et al. recommended avoiding ASCT in elderly patients with significantly compromised renal function unless it is clearly related to active myeloma that is unresponsive to other therapy [22]. Certain treatment-related toxicities are more common in the older adults, when compared with younger patients, and commonly include cardiac arrhythmias (8% vs. 0%) and GI toxicity (68% vs. 46% grade 3-4 diarrhea; 45% vs. 23% grade 3-4 oral toxicity) [36, 37]. Comorbidities are associated with greater toxicity and increased length of hospital stay.

Effectiveness of HDT plus ASCT in elderly patients

In the absence of randomized studies on the efficacy of ASCT in elderly patients with MM, several cohort studies have compared the response rates, PFS and OS with ASCT between older and younger patients. An Italian study has proposed two to three courses of melphalan, 100 mg/m2, supported by ASCT [38] and demonstrated that this approach was superior to conventional chemotherapy with MP in patients aged 50 to 70 years, including patients aged 65 to 70 years (44 in 4). Another trial, the Intergroupe Francophone du Myelome (IFM) 99-06, reported conflicting results regarding the benefit of ASCT over conventional therapy in older adults. In the IFM 99-06 trial, patients aged 65-75 years were randomly assigned to MP alone, MPT, or induction chemotherapy followed by two courses of melphalan (100 mg/m2) with ASCT [11]. In this trial, the MPT regimen yielded a higher response and CR rate than MP and was significantly superior both to MP and to melphalan plus ASCT.

In a post hoc analysis of a phase II study, the subgroups of patients older than age 65 years who underwent ASCT were compared with those who did not [39]. Both groups received thalidomide-based induction and transplant ineligible patients received two additional cycles of chemotherapy followed by thalidomide maintenance. The ASCT group had higher response rates; however, there was no benefit in PFS and OS when compared to non ASCT group.

The use of novel agents in combination with intermediate-dose melphalan might improve results by increasing the efficacy/toxicity ratio of induction treatment and by delaying relapses with post ASCT consolidation/maintenance. An Italian group studied the combination regimen of bortezomib combined with doxorubicin and dexamethasone (PAD regimen) in a group of 102 patients aged 65 to 75 years (45 in 4). The induction treatment was effective with the rate of CR plus very good partial response (VGPR) 58%. In this study, 90% of patients received the first ASCT and 83% received the second ASCT. Patients received lenalidomide consolidation/maintenance therapy after ASCT, and the final rate of CR plus VGPR increased to 78% with two-year PFS and OS rates of 69% and 86%, respectively.

Challenges in treating elderly multiple myeloma patients

Deciding how aggressively to treat MM in the elderly is a clinical challenge that needs a thorough understanding of indications and possible outcomes of the treatment. The decision to initiate combination chemotherapy should be taken by a multidisciplinary team considering all available diagnostic and clinical information and in discussion with the patient and their family. Comprehensive geriatric assessment (CGA) tools have been created to clinically assess frailty, functional status, comorbidities, cognitive ability, and nutritional status [40-42]. A specific CGA has been developed for assessment of MM patients to help guide treatment decisions in elderly patients [43]. This score was developed by a pooled analysis of data from three clinical trials consisting of 869 patients [33, 43]. The discussion of treatment should include all alternatives, including palliative and complementary approaches. Given the increased rates of disease-related and treatment-related complications in the elderly patients, providing appropriate supportive care is of vital importance.

Special considerations

When considering treatment options for MM in the older adults, certain age-related comorbidities may play a vital role in choosing one regimen over the other. Thalidomide and bortezomib can be used at full doses in the presence of renal dysfunction and with hemodialysis, whereas the dose of lenalidomide needs adjustment. Older people are at increased risk of venous thromboembolism (VTE) because of impaired mobility. Risk of VTE is further enhanced with the use of immunomodulatory agents, thalidomide and lenalidomide in elderly MM patients. Anemia could be exacerbated by the chemotherapy and should be adequately managed with judicious red blood cell transfusion and intravenous iron infusion. Peripheral neuropathy is a common adverse effect of many MM therapies, such as thalidomide and bortezomib. The problem of peripheral neuropathy could be worrisome in MM in patients with diabetes. Elderly patients receiving MM therapy that affects gastrointestinal motility, such as bortezomib, are more prone to develop severe mucositis and diarrhea. Polypharmacy is quite prevalent in the older population. Thus, careful review of the patient’s current list of medications and attention to potential drug-drug interactions is required.

## Conclusions

In conclusion, the availability of new combination regimens and enhanced safety of ASCT has increased the treatment options for elderly patients with multiple myeloma. Combination therapies including novel agents have improved the response and survival rates in transplant-ineligible patients. MPT, MPV, and lenalidomide/dexamethasone represent new standards of care for transplant-ineligible MM patients. It is important to assess each patient’s clinical situation and fitness for therapy to allow treatment to be provided at an appropriate intensity. Particular care must be taken to minimize toxicities, reducing doses if required, to allow continuation of treatment when appropriate. Treating myeloma in the very elderly is challenging, and clinicians should develop a personalized approach to optimize the treatment response while minimizing toxicity through careful geriatric assessment and compassionate care.
